# Updating the German Psycholinguistic Word Toolbox with AI-Generated Estimates of Concreteness, Valence, Arousal, Age of Acquisition, and Familiarity

**DOI:** 10.5334/joc.482

**Published:** 2026-01-08

**Authors:** Javier Conde, Gonzalo Martínez, María Grandury, Carlos Arriaga, Juan Haro, Sascha Schroeder, Florian Hintz, Pedro Reviriego, Marc Brysbaert

**Affiliations:** 1 Information Processing and Telecommunications Center (IPTC), Universidad Politécnica de Madrid, Spain; 2 Universitat Rovira I Virgili. Departament of Psychology and CRAMC, Spain; 3 Institute of Psychology, University of Göttingen, Germany; 4 Research Center Deutscher Sprachatlas, Marburg University, Germany; 5 Department of Experimental Psychology, Ghent University, Belgium

**Keywords:** AI-generated word norms, familiarity, concreteness, valence, arousal, age of acquisition, German language

## Abstract

This article presents AI-generated estimates for five characteristics of German words: concreteness, valence, arousal, age of acquisition (AoA), and word familiarity. The estimates were generated using GPT-4o-mini, which was selected due to its good performance in previous studies. Validation studies were conducted comparing the AI-generated estimates with both human ratings and previously generated AI data to ensure their usefulness for research applications. The main results are as follows. The GPT estimates of word concreteness, valence, and arousal show a strong correlation with human ratings but are not better than the best available AI-generated estimates based on semantic vectors. The GPT estimates of AoA are good approximations of human ratings and outperform other available alternatives (except for human ratings), especially after the model was fine-tuned based on 2,000 human ratings. Fine-tuned AI-generated estimates of word familiarity have better predictive value than word frequency for word recognition in lexical decision tasks and vocabulary tests. Estimates for concreteness, valence, arousal, and AoA are available for 167,000 words, which are likely to be known to more than 90% of participants in typical adult studies. Word familiarity estimates are presented for 928,000 word forms. All data and codes, including newly collected human familiarity ratings for 11,000 words, are publicly available at https://osf.io/ghjd2/. The data may be freely used for research purposes, but not for commercial purposes.

Research across a wide range of scientific disciplines requires information about the properties of words. This need extends from research focusing on word recognition and word production —where the characteristics of words are the primary subject of study — to broader experimental designs that use matched lexical items.

The primary word-related variables are word length, word frequency or familiarity, orthographic/phonological similarity to other words, age of acquisition (AoA), concreteness, valence, and arousal. While some of these properties (e.g., word length, frequency, similarity) can be calculated from dictionaries or language corpora, others, such as familiarity, AoA, concreteness, valence, and arousal, depend on subjective judgments and require extensive norming studies. A major challenge in many languages, including German, is the limited and fragmented availability of such psycholinguistic norms.

Advances in natural language processing gradually introduced tools to approximate subjective human judgments. A first breakthrough was the use of word vectors to estimate values for yet-to-be-normed words based on a seed sample of normed words. Word vectors are high-dimensional, numerical representations of words that capture their semantic and syntactic relationships based on co-occurrence patterns in large text corpora.

The use of semantic vectors yielded estimates that correlated encouragingly with human norms. Bestgen and Vincze (2012) were among the first to demonstrate this by estimating values for non-normed words based on the similarity of their semantic vectors to those of normed words. While these correlations, ranging from .59 to .79, were promising, they were not yet sufficient for detailed investigation (Mandera et al., 2015). Accuracy improved when researchers switched from averaging values of words with similar semantic vectors to directly regressing the semantic vectors on human norms (Hollis et al., 2017) or even norms collected in another language (Thompson & Lupyan, 2018). This allowed the calculation of useful approximations for thousands of words in tens of languages.

Further progress was made with the introduction of large language models (LLMs). LLMs are machine learning systems trained on huge amounts of text to predict the next word, taking into account larger word dependencies than a window of a few words before and after each target word, typical for semantic vectors (Hussain et al., 2024). Researchers found that even without a set of norms as a starting point, such models could produce valid estimates of subjective word characteristics. This was first shown for English, the language in which the models were mostly trained (Brysbaert et al., 2025; Heyman & Heyman, 2024; Martínez, Molero, et al., 2025b; Trott, 2024). Martínez et al. (2025a) and Sendín et al. (2025) subsequently reported good data for Spanish as well.

Here we present and evaluate LLM-generated information for German words. The variables are word concreteness, valence, arousal, AoA and familiarity. The LLM model we used was gpt-4o-mini-2024-07-18. It has the disadvantage of being a commercial product (openai.com) that does not guarantee longevity or information about what went into the model, but it is a model that currently gives better estimates than open models (Conde et al., 2025a). This aligns with our goal of providing colleagues with a time-stamped list of the best possible AI-generated estimates of psycholinguistic variables, rather than trying to understand how LLMs generate such values. The goal is similar to collecting and validating lists of human ratings, about which we also have little information on how they are generated.

Nine studies will be discussed.^1^ Study 1 investigates the quality of concreteness estimates; Study 2 that of the valence and arousal estimates. Study 3 examines the quality of age of acquisition estimates. The last six studies look at the quality of AI-generated estimates of word familiarity by correlating the estimates with several validation criteria and by collecting new sets of human familiarity ratings. All code and data can be found at https://osf.io/ghjd2/.

## Study 1: Evaluating the GPT concreteness estimates

Word concreteness was one of the first variables for which AI-generated estimates were developed (Bestgen & Vincze, 2012; Hollis et al., 2017; Thompson & Lupyan, 2018). Given that word concreteness is very similar in different languages, certainly in English and German (Thompson & Lupyan, 2018), good AI-generated estimates were expected for this variable. It is an important word characteristic that is often required for research purposes, for example to investigate whether brain activity differs when processing abstract versus concrete words (Pauligk et al., 2019).

An advantage of GPT is that working with the model does not require much specialized knowledge. To obtain independent and reproducible estimates, it is advisable to work with an application programming interface (API) called from a Python or R program rather than a web chat interface, but these are skills that many researchers have or can develop (Conde et al., 2025b).

The quality of LLM-generated estimates also depends on the prompt used (Conde et al., 2025b). We used the following prompt, based on our previous experiences. It included three examples of words with low concreteness (essentiality, although, hope) and three examples of high concreteness words (bat, marzipan, blackbird) to calibrate the output.

Bitte bewerten Sie die Konkretheit des folgenden Wortes auf einer Skala von 1 bis 7, wobei 1 für sehr abstrakt und 7 für sehr konkret steht. Beispiele für Wörter, die eine Bewertung von 1 erhalten würden, sind *Wesentlichkeit, obwohl*, und *Hoffnung*. Beispiele für Wörter, die eine Bewertung von 7 erhalten würden, sind *Fledermaus, Marzipan* und *Amsel*. Das Wort lautet: [Wort hier einfügen]. Geben Sie für dieses Wort nur eine Zahl zwischen 1 und 7 an. Bitte beschränken Sie Ihre Antwort auf Zahlen.

The prompt was repeated before each word to prevent response dilution. Temperature was set to 0. Using this method, the estimates from two different runs of the model with different word order correlate with each other by more than 0.96, so it is not necessary to run the model multiple times and average the estimates.^2^ We used the logprobs output, which gives the probability of the possible answers, from which we could calculate a more detailed estimate based on the weighted average of the different possibilities provided by the LLM (for further information, see Martinez et al., 2025b).

There are several databases with which we could compare the German GPT concreteness estimates. First, there are three studies that collected human ratings of concreteness. They are in chronological order:

1. Lahl et al. (2009): These authors collected human concreteness ratings for 2,654 nouns on an 11-point Likert rating scale.^3^
2. Kanske & Kotz (2010): Human concreteness ratings for 1000 nouns on a 9-point Likert rating scale. This scale was reverse coded as high numbers indicated more abstract words, but was brought in line with the others for the present analysis.
3. Charbonnier & Wartena (2020) combined concreteness ratings collected by Baschek et al. (1977) and Wippich & Bredenkamp (1979) for 1698 words with those of Lahl et al. (2009) and Kanske and Kotz (2010) and recalculated them on a 7-point rating scale. In total they provided ratings for 4,182 German words.

The second source of information consists of four studies in which human ratings of imageability were collected. Imageability (how easily can you form a mental image of the concept represented by the word?) is not exactly the same as word concreteness, but correlates highly with it, given that concreteness ratings are strongly dominated by the visual modality (Lynott et al., 2020). The four studies are:

1. Võ et al. (2006) collected imageability ratings for 2902 words, using a 7-point imageability scale ranging from 1 (low imageability) to 7 (high imageability).
2. Schmidtke et al. (2014) collected imageability ratings for 1034 words on a nine-point rating scale.
3. Schröter & Schroeder (2017) collected imageability ratings for 1152 words on a seven-point Likert scale.
4. Grandy et al. (2020) collected Imageability ratings for 2500 nouns from a group of young (21–31 yrs old) and old adults (70–86 yrs old) on a sliding scale from 0 to 100.

We also found three large-scale sets of AI estimates based on semantic vectors:

1. Köper & Schulte im Walde (2016) provided concreteness and imageability estimates for 350,000 lemmatized words based on semantic vectors and linear regression analysis with the German ratings of Lahl et al. (2009) and Kanske & Kotz (2010), and with ratings of translated English words.
2. Thompson & Lupyan (2018) provided estimates for one million German words based on semantic vectors and linear regression with the ratings of translated Dutch and English words.
3. Lüdtke & Hugentobler (2022) obtained estimates for concreteness and imageability for 933, 813 words based on semantic vectors and linear regression analysis with the values calculated by Charbonnier & Wartena (2020).

We collected GPT estimates for 185 thousand words, based on initial GPT_FAM estimates above 3.5 (from Study 4). Brysbaert et al. (2025) observed that words with lower GPT_FAM estimates are unlikely to be known to 90% of student participants in psychology experiments and, therefore, to be of interest for studies on concreteness.

**Figure 1:**
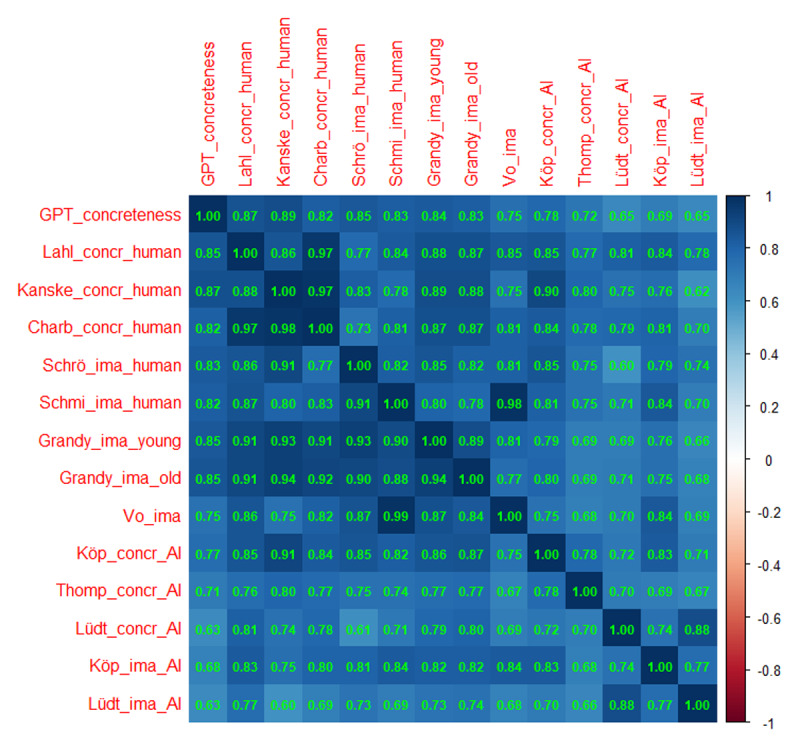
Correlations of the GPT estimates with the various other measures of concreteness and imageability. Above the diagonal: Spearman correlations; below the diagonal: Pearson correlations. Notice that the number of data pairs differs per cell, depending on the size of the datasets involved. Minimal number of data pairs for correlations between AI-generated estimates and human ratings was 900, minimal number of correlations between AI-generated estimates was 70,000.

Figure 1 shows the Spearman and Pearson correlations between the various measures. We prefer Spearman correlations because they are less affected by outliers and give more weight to differences around the mode (Conde et al., 2025a). For most correlations reported here, the Spearman coefficient is slightly higher than the Pearson coefficient.

The human ratings correlated highly with each other, also the ones between concreteness and imageability. The GPT-estimates were also highly correlated with the human concreteness ratings (r > .82) and seemed to be on par with the concreteness and imageability estimates of Köper and Schulte im Walde (2016).

**Figure 2:**
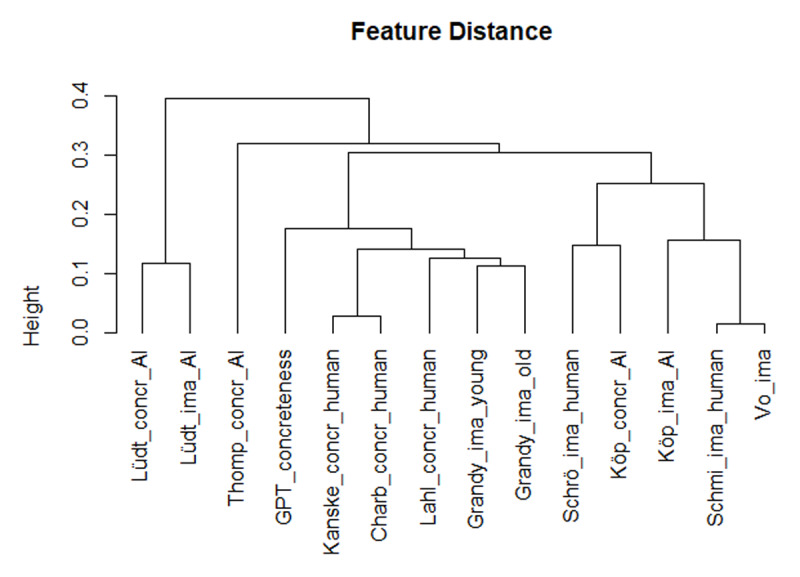
Hierarchical cluster analysis based on Spearman correlations, showing that the GPT concreteness estimates correlated closely with the human concreteness ratings (based on corr_cluster from the AnalysisLin R package; Lin, 2024).

The comparison of the various measures becomes easier if we use a hierarchical cluster analysis, which groups the variables as a function of their overall similarity. Figure 2 shows the tree diagram based on the Spearman correlations. It shows that the GPT concreteness ratings were closer to the human concreteness ratings, whereas the Köper and Schulte im Walde (2016) estimates tended to be closer to the imageability ratings. The other AI measures were more distant and, hence, less useful as proxy for human ratings.

## Study 2: Valence and arousal

Valence and arousal are two other important word variables in research (e.g., Bayer et al., 2010) for which useful AI-generated estimates have been obtained (Bestgen & Vincze, 2012; Hollis et al., 2017). Therefore, we collected information about these variables as well. The prompt we used for valence was:

Bitte bewerten Sie, wie sich eine Person fühlt, wenn sie das folgende Wort liest. Verwenden Sie eine Skala von 1 bis 7, wobei 1 sehr negativ, schlecht und 7 sehr positiv, gut bedeutet. Beispiele für Wörter, die eine Bewertung von 1 erhalten würden, sind *Pädophiler, AIDS* und *Wrack*. Beispiele für Wörter, die eine Bewertung von 7 erhalten würden, sind *Urlaub, fantastisch* und *lachen*. Das Wort lautet: [Wort hier einfügen]. Geben Sie für dieses Wort nur eine Zahl zwischen 1 und 7 an. Bitte beschränken Sie Ihre Antwort auf Zahlen.

The prompt for arousal was:

Bitte bewerten Sie, wie sich eine Person fühlt, wenn sie das folgende Wort liest. Verwenden Sie eine Skala von 1 bis 7, wobei 1 für sehr ruhig und entspannt und 7 für sehr erregt und aufgeladen steht. Beispiele für Wörter, die eine Bewertung von 1 erhalten würden, sind “Getreide”, “langweilig” und “Ruhe”. Beispiele für Wörter, die eine Bewertung von 7 erhalten würden, sind “Waffe”, “Liebhaber” und “Nervenkitzel”. Das Wort lautet: [Wort hier einfügen]. Geben Sie für dieses Wort nur eine Zahl zwischen 1 und 7 an. Bitte beschränken Sie Ihre Antwort auf Zahlen.

There were six human datasets we could use to validate the GPT estimates. In chronological order they are:

1. Lahl et al. (2009) collected valence and arousal ratings for 2654 German nouns on an 11-point rating scale ranging from 0 to 10.
2. Kanske and Kotz (2010) collected valence and arousal ratings for 1000 nouns on a 9-point rating scale.
3. Schröter and Schroeder (2017) collected valence and arousal ratings for 1152 words. Valence was measured with a 7-point scale, ranging from –3 to +3. Arousal was measured with a 5-point scale from 1 to 5.
4. Schmidtke and Conrad (2018) grouped valence and arousal ratings for 5695 words. Most of the ratings had been described in two previous publications (Schmidtke et al., 2014; Võ et al., 2009). The same scales were used as in Schröter and Schroeder (2017).
5. Grandy et al. (2020) collected valence ratings for 2592 German nouns from 20 younger and 20 older adults on a sliding scale from 0 to 100. No arousal measures were collected.
6. Xu et al. (2025) collected valence and arousal ratings from Chinese-German bilinguals for 880 words from the Schmidtke and Conrad (2018) list. The instructions for valence were the same as in Schmidtke and Conrad; for arousal a 7-point ratings scale was used as well, going from 1 to 7.

In addition, there were two AI-generated datasets.

1. Köper & Schulte im Walde (2016) provided valence and arousal estimates for 350,000 lemmatized words based on semantic vectors and linear regression analysis with the German ratings of Lahl et al. (2009), Võ et al. (2009) and Kanske & Kotz (2010).
2. Lüdtke & Hugentobler (2022) obtained estimates for valence and arousal for 933,813 words based on semantic vectors and linear regression analysis with the BAWL-R ratings.

**Figure 3:**
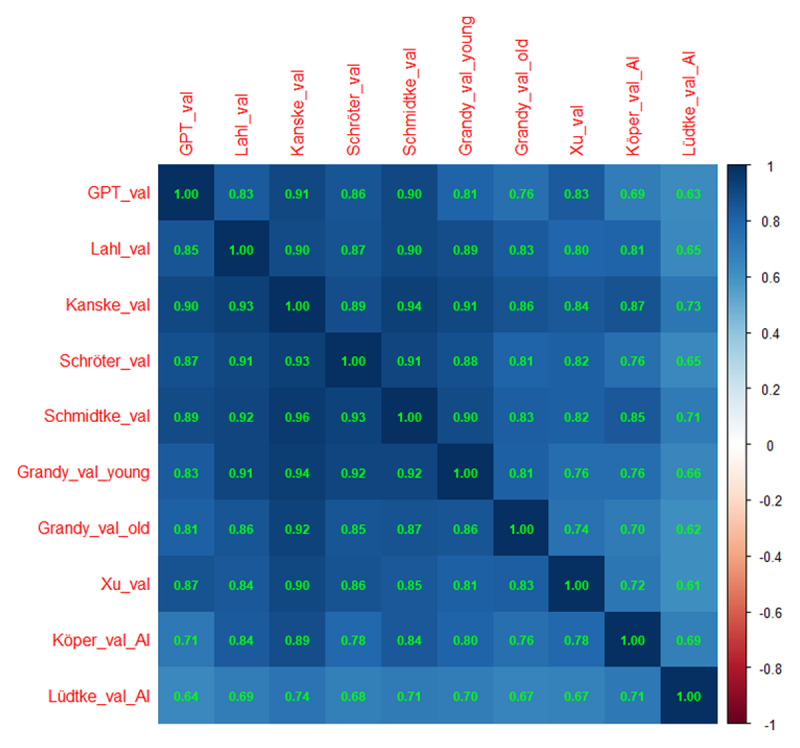
Correlations of the GPT estimates with the various other measures of valence. Above the diagonal: Spearman correlations; below the diagonal: Pearson correlations. Notice that the number of data pairs differs per cell, depending on the size of the datasets involved. Minimal number of data pairs for correlations between AI-generated estimates and human ratings is 880, minimal number of correlations between AI-generated estimates is 70,000.

**Figure 4:**
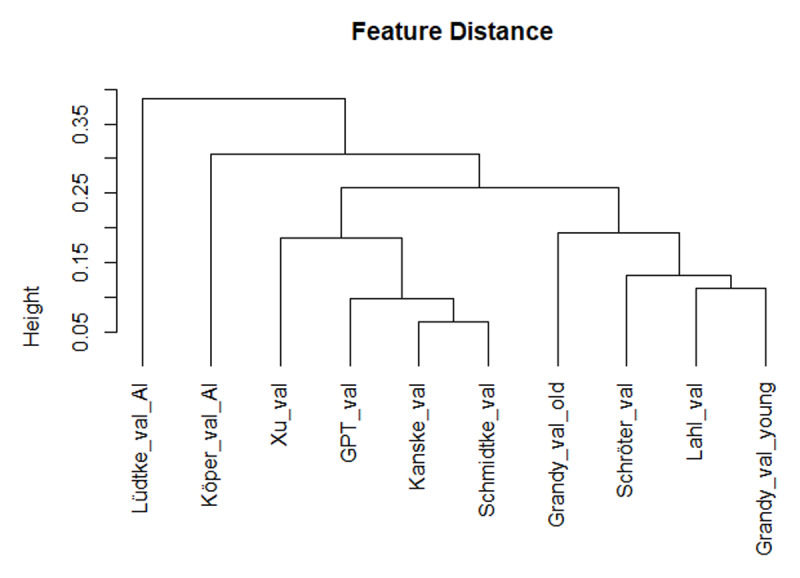
Hierarchical cluster analysis based on the Spearman correlations, showing that the GPT valence estimates correlate closely with the human valence ratings (based on corr_cluster from the AnalysisLin R package; Lin, 2024).

Figures 3 and 4 show the correlations and the hierarchical cluster analysis for valence. All human ratings correlated more than .80 with each other. Also the correlations of the GPT estimates with human ratings were r > .75, with most above .8. Correlations of the GPT estimates with the other AI estimates were lower. Of those, Köper & Schulte im Walde (2016) correlated most with the human ratings. The hierarchical cluster analysis confirmed that both the GPT estimates and the estimates of Köper & Schulte im Walde (2016) gave close approximations to the human ratings.

**Figure 5:**
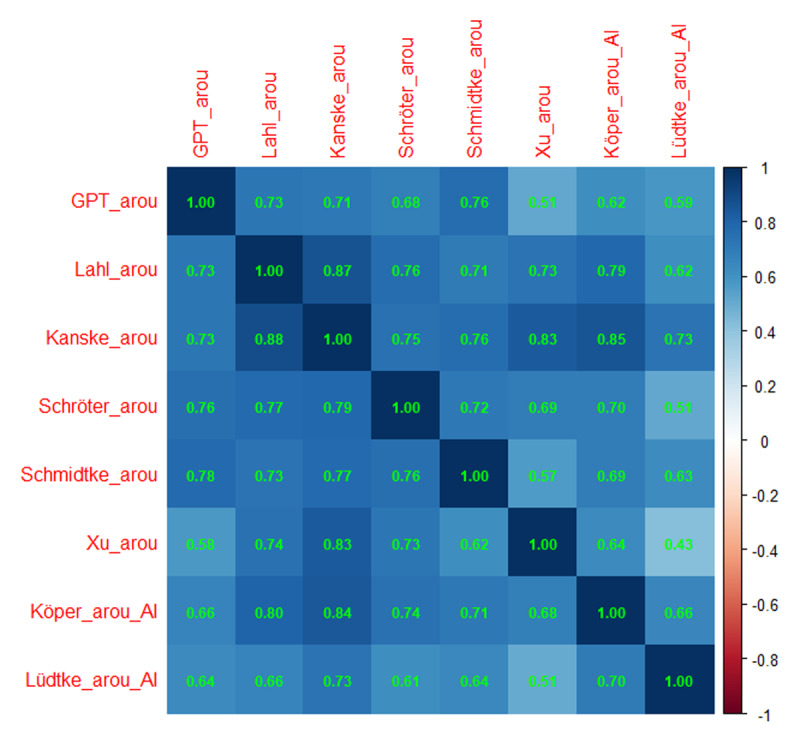
Correlations of the GPT estimates with the various other measures of arousal. Above the diagonal: Spearman correlations; below the diagonal: Pearson correlations. Notice that the number of data pairs differs per cell, depending on the size of the datasets involved. Minimal number of data pairs for correlations between AI-generated estimates and human ratings is 880, minimal number of correlations between AI-generated estimates is 70,000.

**Figure 6:**
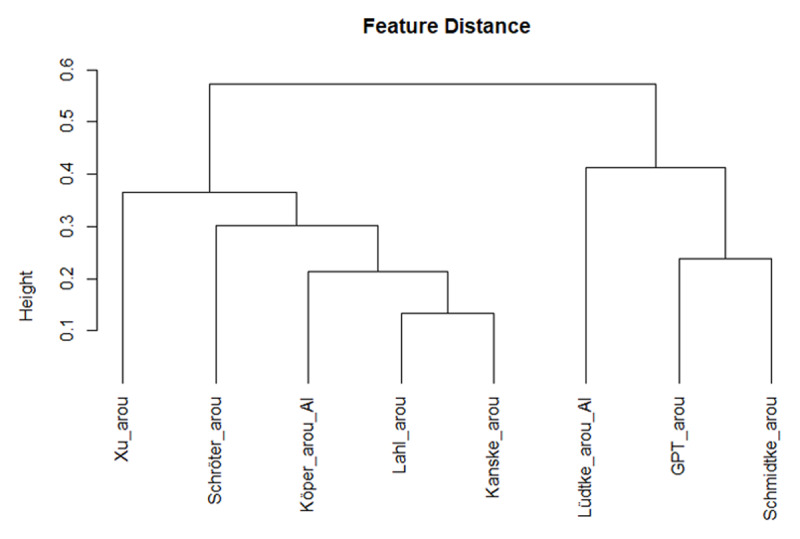
Hierarchical cluster analysis based on the Spearman correlations, showing that the GPT estimates of arousal tend to be less correlated with the human data than the AI estimates of Köper & Schulte im Walde (2016) (based on corr_cluster from the AnalysisLin R package; Lin, 2024).

Figures 5 and 6 give the information for arousal. As has been found in other languages (Martinez, Conde et al., 2025a; Martinez et al., 2025b), the correlations between the GPT estimates and human ratings are lower for arousal. This is also true for the correlations between the different studies and may indicate that arousal is an ambiguous concept that will need further refinement (Smith et al., 2025). It does look, however, as if the GPT estimates did slightly worse than the AI estimates of Köper & Schulte im Walde (2016), given that the latter correlated more with the human ratings and are more central in the hierarchical cluster analysis.

## Study 3: Age of Acquisition

A fourth variable for which it is interesting to have German AI-generated estimates is age of acquisition (AoA), the age at which words are typically acquired. This variable has been shown to influence word processing over and above word frequency (Brysbaert & Ellis, 2016). It is also an important variable in developmental studies (e.g., Grosse et al., 2021).

We used the following prompt:

Das Erwerbsalter eines Wortes bezieht sich auf das Alter, in dem ein Wort zum ersten Mal gelernt wurde. Genauer gesagt, wann eine Person dieses Wort zum ersten Mal verstanden hätte, wenn jemand es vor ihr verwendet hätte, auch wenn sie es noch nicht gesprochen, gelesen oder geschrieben hatte. Schätzen Sie das durchschnittliche Alter, in dem das Wort “{Wort}” von einem deutschen Muttersprachler erworben wurde. Das Ausgabeformat muss ein JSON-Objekt sein. Beispiel: {Wort: {Wort}, Erwerbsalter: //Erwerbsalter des Wortes in Jahren, muss zwei Dezimalstellen haben}

We generated AoA estimates for 202,452 words that were likely to be of interest. These consisted of the 185K words used for concreteness, valence, arousal, plus new words from the datasets described below.

There were four human datasets and one AI-based set with which we could compare the estimates:

1. Birchenough et al. (2017), who collected AoA ratings for 3259 German words.
2. Schröter & Schroeder (2017), who collected human AoA ratings for the 1152 words encountered before.
3. Translation of English words for which Kuperman et al. (2012) collected human AoA ratings. The stimuli were limited to those words for which DeepL forward and backward translation resulted in the same word, thereby excluding translation inconsistencies (N = 18,447).
4. Translation of Dutch words for which Brysbaert et al. (2014) collected human AoA ratings, again limited to the words with consistent forward and backward translations (N = 12,966).
5. Botarleanu et al. (2024) calculated age of exposure estimates (AoE) for more than 50 thousand German words. The underlying idea was to simulate a language learner’s exposure to words via training word vector models on incrementally increasing corpora of texts, so that the model “learned” word meanings by increasing exposure.

**Figure 7:**
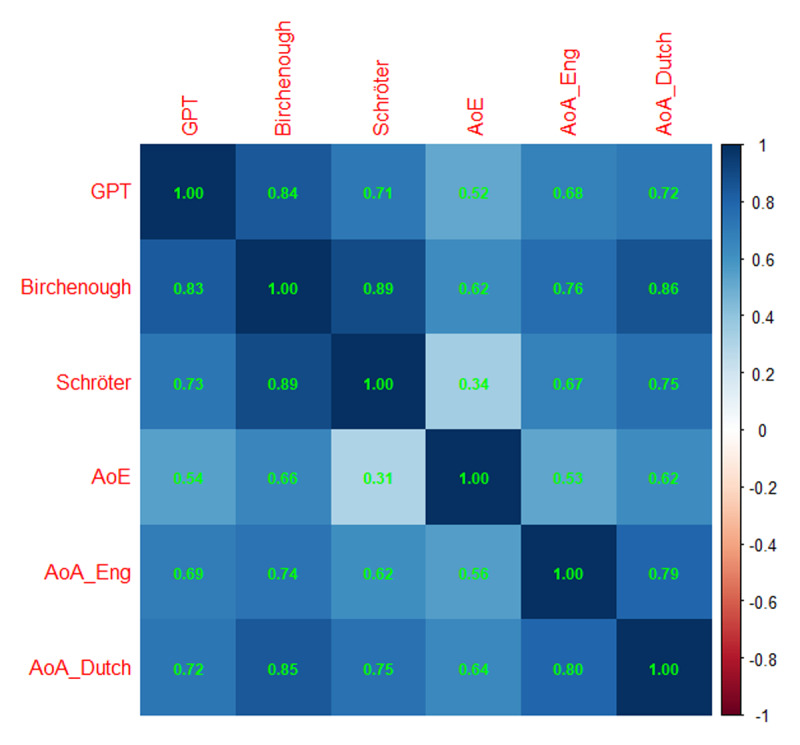
Correlations of the GPT estimates with the various other measures of AoA. Above the diagonal: Spearman correlations; below the diagonal: Pearson correlations. Notice that the number of data pairs differs per cell, depending on the size of the datasets involved. Minimal number of data pairs for correlations between AI-generated estimates and human ratings is 497, minimal number of correlations between AI-generated estimates is 12,900.

Figure 7 shows the outcome. The correlations between the GPT estimates and the human ratings were encouraging, even those with the AoA ratings based on translations from English and Dutch words. Especially the correlation with the Birchenough et al. (2017) ratings was high (ρ = .84, N = 3255). The correlation with the Schröter and Schroeder (2017) ratings was lower (ρ = .71, N = 1149). The correlations of the GPT estimates with the human ratings were higher than those of the AoE estimates with the human ratings, indicating that the GPT estimates are a clear improvement over the AoE estimates.

At the same time, we saw that the correlations between the GPT estimates and the human ratings were lower than the correlation between the human ratings, including the translated ratings.

Sendín et al. (2025) observed that Spanish AoA estimates generated with GPT-4o-mini correlated better with human ratings when the model was fine-tuned on 2000 human ratings. Fine-tuning is a technique that is used to bring the output of an LLM more in line with what is desired (Ouyang et al., 2022; Zhao et al., 2024). It is typically used to make the interaction with a LLM more user-friendly or less biased by unwanted patterns in the training material (e.g., related to group differences).

In the fine-tuning used by Sendín et al. (2025), the model was first asked to give an estimate of AoA and was then provided with feedback about the human rating. Such feedback allowed the model to update its weights, so that the output approximated the human example better. It is not known where in the model the weights change, but an educated guess is that it happens in the later layers of the model, where the information within the model is translated to the expected output.

To see whether fine-tuning would improve the German estimates of AoA as well, we used a random sample of 2000 words from Birchenough et al. (2017) to fine-tune gpt-4o-mini-2024-07-18. The same prompt as with humans was used. After the model gave its estimate, corrective feedback was given in the form of the human rating with two-digit precision (4.21). We used OpenAI’s default settings for updating the weights in the model, which automatically select parameters based on the dataset (see Conde et al., 2025b, for a hands-on tutorial on how to proceed). For the present dataset, it selected 3 epochs, a batch size of 4, and a fixed learning rate of 1.8. We obtained a training loss of 1.047.

**Figure 8:**
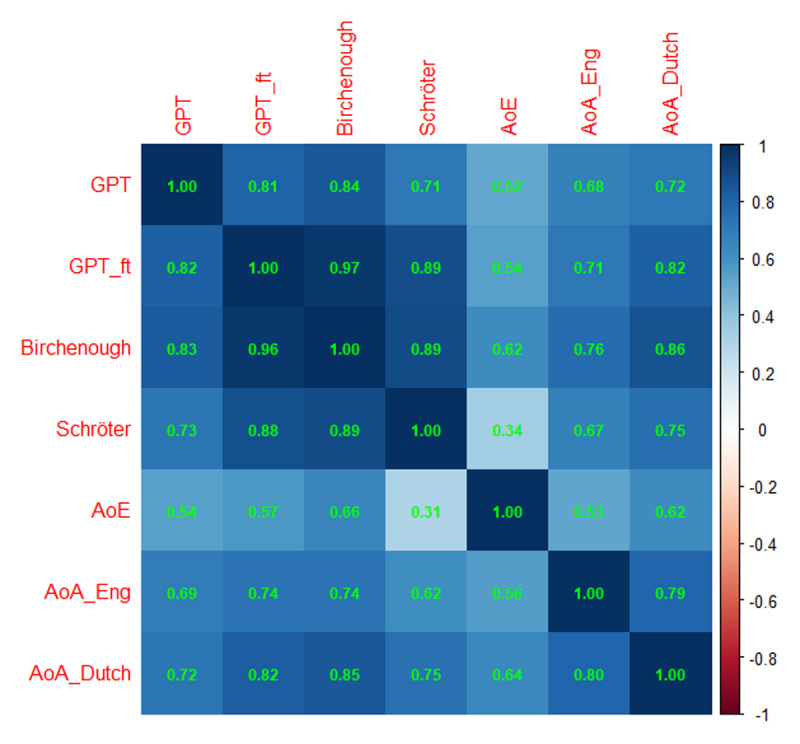
GPT_ft was added as an extra variable to Figure 7, showing the higher correlations between the fine-tuned GPT estimates and the other values than between the original GPT estimates and the other values.

It was not possible to download the fine-tuned GPT model (just like it is not possible to download the original model), but it could be saved on the servers of OpenAI and used for subsequent queries. We used the fine-tuned model to generate new AoA estimates for the 202,452 words we used before. Figure 8 shows the outcome.

As in Spanish, we observed higher correlations between the fine-tuned GPT estimates and the human ratings than between the original GPT estimates and the human ratings. The difference was largest for the Birchenough et al. (2017) ratings, which was largely due to the fact that two thirds of these ratings were used for the fine-tuning. However, also for the 1259 untrained words we saw an increase in correlation to ρ = .93. So, the training was not confined to the words that had been used for feedback; it generalized to the remaining words. We also saw higher correlations with the Schröter and Schroeder (2017) ratings and with the translated ratings.

**Figure 9:**
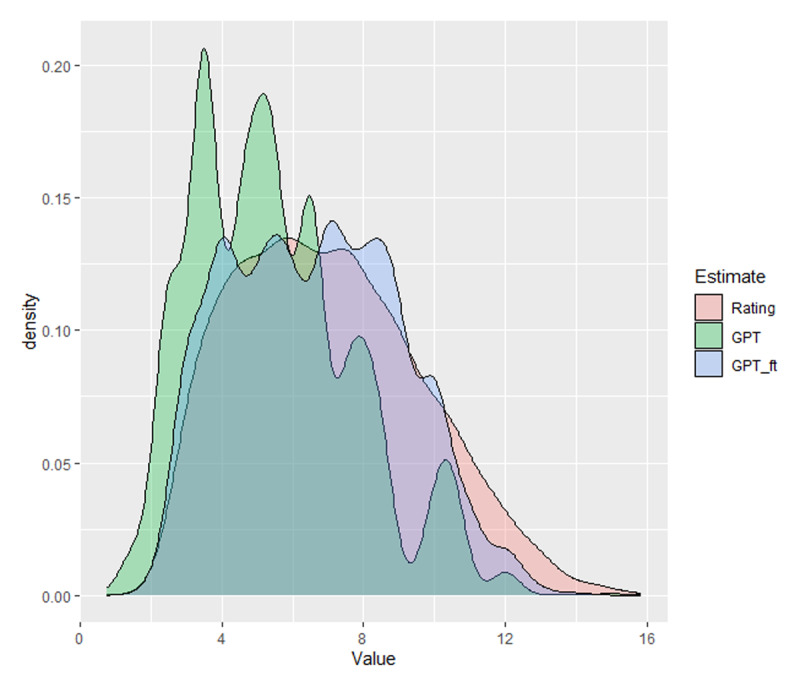
Distribution of the AoA values for human ratings, GPT estimates, and GPT fine-tuned estimates.

Fine-tuning not only increased the correlations between the AI estimates and the human ratings but also brought the distribution of estimates closer to that of the human ratings, as can be seen in Figure 9. Whereas the untrained model gave too many low estimates, the fine-tuned model better aligned with the human ratings.

## Study 4: Word familiarity

In the studies to follow, we investigated the extent to which GPT’s estimates of word familiarity correlate with human data. Of the five variables tested, familiarity was expected to be the most challenging, as it relates to word form (the lexical level) rather than meaning (the semantic level). Therefore, patterns observed in English (the language predominantly used to train GPT) may not transfer to German. They may even interfere.

Brysbaert et al. (2025) and Martínez et al. (2025a) introduced AI-based estimates of word familiarity. They argued that familiarity is a more informative AI-generated variable than word frequency because word frequency either provides no new information (since frequencies of word forms in a representative corpus can be easily counted) or there is ambiguity in the requested information. The latter is true in German for two main reasons. The first is that nouns, verbs and adjectives have different inflections. The second is that compound words are written as single words in German. Take the noun “Haus” (house). It appears 10,265 times in the SUBTLEX-DE corpus of 25.4 million words (Brysbaert et al., 2011). However, the noun has several inflected forms with fairly high frequencies. The most frequent is Hause (from expressions such as “nach Hause,” “zu Hause”), which occurs 11,174 times in the corpus. Other forms are Häuser (572), Hauses (379), Häuschen (145), Häusern (123). Additionally, the word is part of a large number of transparent compounds, such as Krankenhaus (hospital, 2929 times in the corpus), Hausausgaben (homework, 380), Hausmeister (house master, 243), Hausarrest (house arrest, 178), Haushalt (household, 124), Irrenhaus (madhouse, 93), Gasthaus (boarding house, 92), Kaufhaus (department store, 65), and so on.

It is unclear what the answer to the Haus frequency question should be: the frequency of the word form, the frequency of all inflections (called lemma frequency), or the frequency of inflections and transparent compounds (word family frequency)? Similarly, it is unclear what the best frequency answer is for Häuser (houses). Asking how familiar someone is with the word circumvents this problem and provides new information on top of frequency counts in corpora. Indeed, word familiarity estimates have been presented by several authors as a better measure of word knowledge than frequency counts (Balota et al., 2001; Chen & Dong, 2019; Gernsbacher, 1984), although others have objected that it is a catch-all variable including many different factors influencing word processing (e.g., Cordier & Le Ny, 2005; Westbury, 2014).

In English and Spanish, AI-generated familiarity estimates were a significantly better predictor of word knowledge (as measured with response accuracy in a lexical decision task or word selection in a vocabulary test) than word frequency. Brysbaert et al. (2025) found that English AI-estimates of word familiarity not only correlated with word frequency but also with word AoA and word prevalence (how many people know the word). Further reasons for the good performance of AI-generated familiarity estimates may be that it also captures aspects of semantic richness (Yap et al., 2012) and morphological transparency (Jared et al., 2017). As such, AI-generated estimates of familiarity seem to be a catch-all variable that is particularly useful for practical purposes such as word selection and word matching.

### Human familiarity rating studies in German

It turned out to be very difficult to find large-scale familiarity ratings of German words. Instead, we had to resort to four studies that measured related features. In chronological order they are:

1. Schröder et al. (2012) collected concept familiarity ratings for 824 exemplars of 11 semantic categories, including natural categories (animals, birds, fruits, and vegetables), man-made categories (clothing, furniture, vehicles, tools, and musical instruments), as well as professions and sports. Participants were asked to estimate the degree to which they thought about or came in contact with a concept, using a 5-point scale ranging from 1 (very unfamiliar) to 5 (very familiar). Care was taken to make sure that the estimate was attributed to the concept and not to the word.
2. Schröter & Schroeder (2017) estimated the subjective frequency of 1152 words in spoken and written German. Norms were based on a rating study conducted with 100 German university students, who rated the use and occurrence of a word on a seven-point Likert scale ranging from 1 (never) to 7 (several times a day).
3. In LinguaPix (Krautz & Keuleers, 2022) familiarity ratings were obtained for 1248 color photographs of objects with good name agreement. Familiarity was understood as the degree of how usual or unusual the photographed item was in the realm of the participant’s experience (1 – unfamiliar, 6 – familiar).
4. Xu et al. (2025) collected familiarity ratings for 880 German words according to Chinese participants who learned German as a second language. Participants were given a 7-point Likert scale going from 1 (least familiar) to 7 (most familiar).

Together, the four datasets contained information about 3195 unique words, for which we collected AI-generated familiarity estimates.

### Word frequency

Word familiarity is related to the frequency with which words occur in the language. Word frequency is estimated by counting words in representative language samples, called corpora. We used three frequency measures:

1. SUBTLEX-DE (Brysbaert et al., 2011): Word frequency counts based on subtitles from 4,610 films and television episodes, with a total of 25.4 million words.
2. Multilex: A newly created frequency measures obtained by combining the subtitle frequencies of van Paridon & Thompson (2021; corpus size = 139,270,380), with the WorldLex frequency (Gimenes & New, 2016) for blogs (corpus size = 18,428,702), tweets (corpus size = 17,667,011) and newspapers (corpus size = 19,491,446). Frequencies are expressed as Zipf values (log10 frequency per billion words).
3. ChildLex (Schroeder et al., 2015): Word frequencies based on children’s books (primary school) for a total corpus size of 10 million words. This dataset gives lemma frequencies in addition to word form frequencies. Lemma frequencies are the sum of the frequencies of all inflected forms of a lemma (e.g., the sum of the word frequencies: play, plays, played, playing).

All frequencies were log-transformed to take into account that the difference between frequency 1 and 2 has the same impact as the frequency difference between 100 and 200.

### Prompt used

Familiarity estimates were obtained with the following prompt, based on the instructions used for the Glasgow norms (Scott et al. 2019):

Führen Sie die folgenden Aufgaben als deutscher Muttersprachler aus. Vertrautheit ist ein Maß dafür, wie vertraut etwas ist. Ein deutsches Wort ist sehr VERTRAUT, wenn man es oft sieht/hört und es leicht erkennbar ist. Im Gegensatz dazu ist ein deutsches Wort sehr UNVERTRAUT, wenn man es selten sieht/hört und es relativ schwer zu erkennen ist. Bitte geben Sie auf einer Skala von 1 (SEHR UNBEKANNT) bis 7 (SEHR BEKANNT) an, wie bekannt Sie jedes deutsche Wort finden, wobei die Mitte für eine mittlere Bekanntheit steht. Das deutsche Wort lautet: [hier ein Wort einfügen]. Geben Sie nur eine Zahl zwischen 1 und 7 an. Bitte beschränken Sie Ihre Antwort auf Zahlen.

As there were some indications that GPT-4o-mini may give equally useful familiarity information for a non-English language if the instructions are in English (Q. Cai, personal communication), we also used the following prompt:

Complete the following tasks as a native speaker of German. Familiarity is a measure of how familiar something is. A German word is very FAMILIAR if you see/hear it often and it is easily recognisable. In contrast, a German word is very UNFAMILIAR if you rarely see/hear it and it is relatively unrecognisable. Please indicate how familiar you think each German word is on a scale from 1 (VERY UNFAMILIAR) to 7 (VERY FAMILIAR), with the midpoint representing moderate familiarity. The German word is: [insert one word here]. Only answer a number from 1 to 7. Please limit your answer to numbers.

Notice that we did not include exemplars of words with low and high familiarity in the prompts. This is because in the first search we wanted to get the estimates of the standard model, without further help. The prompt was repeated before each word to prevent estimate dilution. Temperature was set to zero.

### Correlations

Table 1 shows the Spearman correlations between the variables. The Pearson correlations can be obtained with the R code at the osf site.

**Table 1:** Spearman correlations between human familiarity ratings, GPT estimates, and word frequency norms. Below the diagonal: number of data pairs on which the correlation is based.


	SCHRöDER_12	SCHRöTER_17	LINGUAPix	XU_25	GPT_GER	GPT_ENG	SUBLTLEX	MULTILEX	CHILDLEX	CHILDLEX_LEMMA

Schröder_12		.76	.45	.42	.67	.64	.42	.42	.43	.46

Schröter_17	116		.37	.46	.55	.53	.65	.68	.46	.53

LinguaPix	213	255		.33	.41	.40	.17	.18	.15	.14

Xu_25	31	387	73		.59	.61	.48	.45	.31	.29

GPT_Ger	820	1152	1248	880		.95	.70	.72	.60	.60

GPT_Eng	820	1152	1248	880	3195		.65	.67	.54	.53

Subtlex	636	1150	1001	880	2769	2769		.98	.72	.77

Multilex	767	1152	1144	880	3042	3042	2768		.72	.80

Childlex	605	1148	984	874	2720	2720	2608	2706		.88

Childlex_lem	605	1148	984	874	2720	2720	2608	2706	2720	


It is important to keep in mind that the numbers of data pairs differ because the datasets vary in how many stimuli they contain. The number of data pairs is greatest for the correlation between Multilex and the GPT estimates (N = 3042; 153 missing frequencies in Multilex) and smallest between Xu et al. (2025) and Schröder et al. (2012), where only 31 words were shared between the two datasets.

There are several patterns in the data. First, it shows the fragmented information present in the available human familiarity ratings. Not only are there few shared words, but the correlations between the ratings were rather low, because the stimuli, instructions and participant groups were different in each study. The highest correlation was between Schröder et al. (2012) and Schröter and Schroeder (2017), where we had a Spearman correlation of .76 (N = 116 words in common).

A second interesting finding is that for three of the four studies, the human familiarity ratings correlated more with the GPT estimates than with the frequency counts. The only exception was Schröter & Schroeder (2017) who asked their participants explicitly to rate the estimated *frequencies* of the words in the language. There we see that the correlations with SUBTLEX and Multilex were higher than with GPT familiarity estimates, confirming that familiarity estimates are not fully the same as frequency estimates.

A third finding is that Multilex and SUBTLEX were doing better than ChildLex, in line with their larger corpus size and the fact that ChildLex is based on materials from primary school. Multilex tended to outperform SUBTLEX, as could be expected given the larger size and larger diversity of the underlying corpus. Given that Multilex almost always did better, we only report this variable in the analyses from now on. Lemma frequencies of ChildLex were somewhat more informative than the word form frequencies.

**Figure 10:**
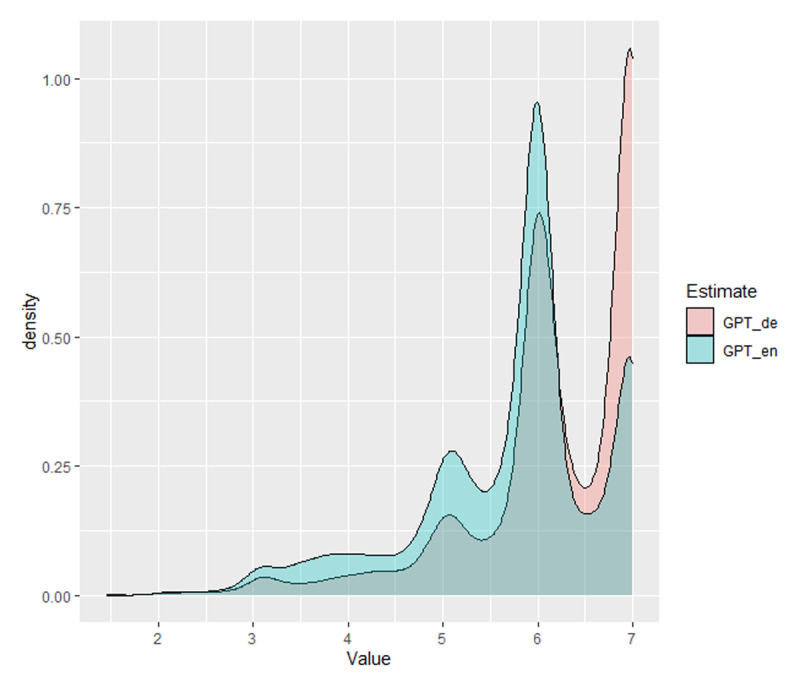
Distribution of the GPT estimates for the German and the English prompt. Most familiarity estimates with the German prompt are around seven, whereas the mode with the English prompt is six.

Finally, there was little difference between the GPT estimates given with the German prompt and with the English prompt. Both correlated .92 with each other. Still, the German prompt slightly outperformed the English prompt on nearly all measures. Figure 10 shows that the familiarity estimates with the English prompts tended to be lower than those with the German prompts. All in all, it looks like good estimates can be obtained with English prompts, but that it is preferable to use a prompt in the language being tested.

## Study 5: New familiarity ratings for words with large differences between GPT familiarity estimates and Multilex word frequency

As the available human familiarity ratings could not be directly compared with the GPT estimates, new sets of ratings were required to assess the validity of the GPT estimates. In the first set, we collected ratings for words whose familiarity estimates differed most from the expected values based on word frequency. As word familiarity and word frequency are often used interchangeably, it is useful to understand what happens when they diverge.

### Stimulus materials

We compiled a list of 2,010 words. These came from a matrix obtained by plotting GPT-FAM estimates for 920,000 words alongside Multilex word frequencies. The 920,000 words came from various sources. First, all stimuli from the various studies discussed in this article were included, except for the Multilex word list, which did not retain the capital letters of nouns. Next, we included a word list with 100,000 lemmas compiled by one of the authors (FH). Such a word list is necessary because some commonly known words do not appear in typical word frequency lists (Schalttag, Kinoticket, Strompreis, Fünfsternehotel, etc.). Finally, we included the 350,000 entries for which Köper and Schulte im Walde (2016) calculated semantic vectors.

**Figure 11:**
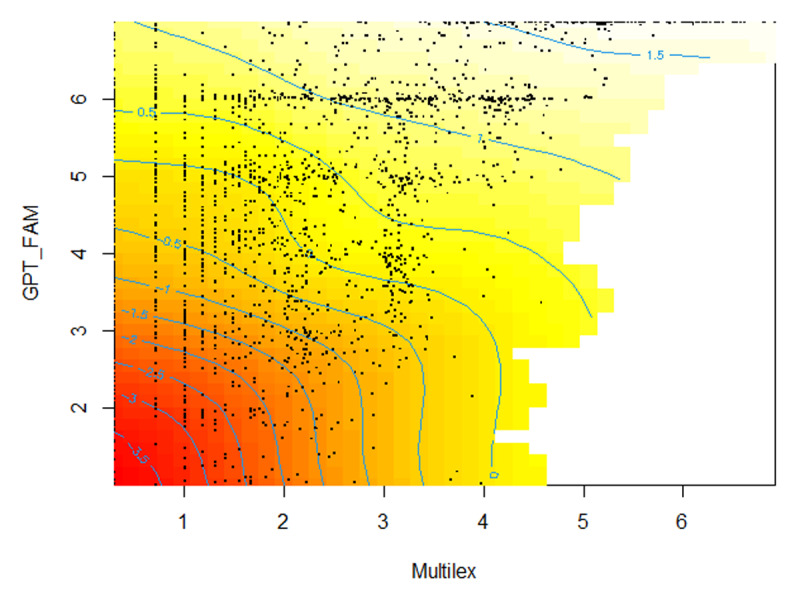
Human ratings as a function of GPT-FAM estimation and Multilex word frequency. Dark red color = low rating, light yellow color = high human rating. Black dots indicate the 2010 words rated. The part of the area without color is a part where there are no stimuli.

The GPT-FAM times Multilex stimulus space was divided into 49 squares (7 × 7), from each of which a random sample of up to 60 words was taken. Figure 11 shows the distribution of the selected words. Notice the empty area in the lower right corner of the curve, as there are no high frequency words with low GPT-FAM estimates. From that area, no or fewer than 60 words per square could be sampled (giving the total of 2010). The selection included words with convergent GPT familiarity and Multilex frequency information (words with high frequency that received high GPT estimates, and words with low frequency that received low GPT estimates) as well as two types of words with divergent information.

The first class of words with divergent information were words with a low GPT familiarity estimate and a relatively high Multilex frequency. There were not many of these words, and a look at them showed that they were mostly taboo words (e.g., related to World War II). Another word was “darren” [to desiccate wood in an oven], a rare word but one with a high Multilex frequency because it is an English name (both first and last name) that appears regularly in English-language films and television series (an important source for word frequency estimates based on subtitles).

The second class of words with divergent GPT familiarity estimates and Multilex frequencies were familiar but rarely occurring words. Most of these were morphologically complex forms of familiar words. There are many such words in the German language, as compound words are written as single words (e.g., “football fanatic” is translated as “Fußballfanatiker”).

### Stimulus presentation and participants

The 2010 stimuli were divided into three lists of 720 words each. Each list contained 75 words, representative of the entire space, that were repeated in all lists, as well as 645 list-specific words (3*645 + 75 = 2010).

The lists were presented via Prolific, a company that provides researchers with access to verified online participants. The familiarity instructions for participants were as follows (based on the instructions for the Glasgow norms; Scott et al., 2019):

Vertrautheit ist ein Maß dafür, wie vertraut etwas ist. Ein Wort ist sehr VERTRAUT, wenn man es oft sieht/hört und seine Bedeutung sehr gut kennt. Im Gegensatz dazu ist ein Wort sehr UNVERTRAUT, wenn man es noch nie gesehen/gehört hat und nicht weiß, was es bedeutet. Bitte geben Sie auf einer Skala von 1 (sehr unbekannt) bis 7 (sehr bekannt) an, wie vertraut Sie mit jedem Wort in der deutschen Sprache sind, wobei die Mitte einen mittleren Vertrautheitsgrad anzeigt.

Wir bitten Sie, 720 Wörter in 12 Blöcken zu je 60 Wörtern zu bewerten. Dies dauert etwa 30 bis 40 Minuten (3 Minuten pro Block). Denken Sie nicht zu lange über Ihre Antwort nach. Folgen Sie einfach Ihrem Gefühl der Vertrautheit.

Wie gut kennen Sie jedes Wort?

**Table:** 

	1	2	3	4	5	6	7	
überhaupt nicht	o	o	o	o	o	o	o	sehr gut

Participants had to click on the number that corresponded to their sense of familiarity with each word. This layout made it unlikely that participants would use AI because it was not possible to download the entire word list and have it rated automatically. The list was divided into 7 blocks of 103 stimuli, so that we could give participants feedback about their progress.

Each list was rated by 21 to 22 participants, with attention paid to gender balance. Participants had to be German speakers, live in Germany, be between 20 and 60 years old, have a 90% approval rating, and have completed at least 10 studies. It took 30 to 40 minutes to complete the list, for which participants received £7. Participants had to agree that their data could be used for scientific research purposes and made available in anonymized form. They were also informed that some of the words were taboo words and had to confirm that they were fine with this.

### Results first familiarity rating study

The data of the familiarity study can be found on the osf website (both the anonymized raw data files and the processed word list).

The reliability of each list was higher than .96 (both Cronbach alpha and McDonald omega) and no participant had to be excluded because of a low correlation with the rest. Participants of the three lists gave an average rating of 5.3 to 5.5 to the common stimuli. Although the difference between the groups was small, we decided to take it into account by calculating best linear unbiased prediction (BLUP) values (Kliegl et al., 2011). These are obtained with a mixed-effects model having random intercepts for stimuli and participants. The stimulus intercepts are the best estimate of word familiarity, based on the full dataset, rather than only the data of the 21–22 participants who responded to a word.

The Spearman correlation between the human ratings and the GPT estimates we collected in Study 1 was ρ = .818 [95% CI: .803–.832], confirming that the GPT estimates are a good proxy for human ratings and that human ratings with the same instructions as given to GPT lead to a higher correlation than the correlations observed in Table 1. For comparison, the correlation of the human familiarity ratings with Multilex was only ρ = .623 [.595–.649]

Figure 11 shows the outcome of a GAM-analysis (Wood, 2001) with GPT familiarity and Multilex frequency as non-linear predictors. The color of the figure gives the human ratings: dark red = low rating, bright yellow = high rating. As could be expected, the lowest ratings were for words both low in GPT familiarity and Multilex frequency. The highest ratings were for words high in GPT familiarity and Multilex frequency.

More important are the estimates for words with divergent information. The most interesting part is the left upper corner, where we have words with low Multilex frequencies and high GPT-FAM estimates. Here we see that the human ratings aligned with the GPT-FAM estimates, as words with low frequency (Multilex < 3 or one occurrence per million words) but high GPT-FAM estimates got high human ratings (yellow color). Hence, the higher correlation of human familiarity ratings with GPT-FAM than with Mulitlex word frequency.

At the same time, the less than perfect correlation between human ratings and GPT-FAM estimates indicates that there are some deviances between both. Table 2 shows the words with the largest differences between the GPT familiarity estimates and the human ratings. The words with higher human ratings than GPT-FAM estimates are nearly all taboo words. The words with lower ratings than GPT-FAM estimates all have very low Multilex frequency, occurring less than once per 10 million words, except for Darren (which is an English name).

**Table 2:** Words with the largest differences between GPT estimates and human ratings. Top: words with lower human ratings than GPT estimates. Bottom: words with higher human ratings than GPT estimates.


WORD	RATING	MULTILEX	GPT_FAM	DIFF

herüben	2.38	1.61	5.73	–3.36

ingeniös	1.83	0.71	5.03	–3.20

wütig	2.85	1.01	5.97	–3.12

dichtbei	1.97	1.01	5.00	–3.03

Matthäuspassion	2.53	1.01	5.05	–2.52

Exonym	1.26	0.30	3.71	–2.45

spitzig	3.71	1.71	5.99	–2.28

darren	1.17	3.45	3.45	–2.28

Sekunda	1.78	0.71	4.01	–2.23

überwach	2.88	1.97	5.03	–2.15

huren	5.97	3.89	2.14	3.83

Endlösung	5.62	2.35	1.39	4.23

Schützenkönig	5.28	1.66	1.04	4.24

Kinderschänder	6.24	2.90	1.90	4.34

Neger	5.42	3.68	1.06	4.35

Ecktisch	5.75	2.05	1.06	4.70

Fotze	6.02	3.73	1.17	4.85

Hitlergruß	6.02	1.66	1.10	4.92

Hurensohn	6.32	3.97	1.01	5.31

Hakenkreuz	6.35	2.71	1.02	5.33


All in all, Study 5 showed that GPT-fam estimates correlated well with human familiarity ratings, but that there were several deviations, which could be improved with fine-tuning in the same way as the AoA estimates were improved in Study 3.

## Study 6: Familiarity ratings for 9000 extra words

The ratings collected in Study 5 revealed systematic deviations between GPT estimates and human familiarity ratings, which could be mitigated through model fine-tuning. Unfortunately, the human ratings collected in Study 5 were insufficient for this purpose because they mainly assessed exceptional cases with significant discrepancies between the untuned GPT estimates and word frequency. What was needed were additional estimates for a more representative sample of the entire familiarity distribution of German words. As we had concerns about the quality of the existing ratings (see Table 1), we decided to collect familiarity ratings for an additional 9,000 words. The number was decided based on two considerations: (1) ideally, we should distinguish between training and testing data, and (2) a larger training sample may be required to obtain familiarity estimates for over 900,000 words (of which 9,000 represent only 1%).

Nearly one third of the 9,000 stimuli were samples from existing familiarity studies (see Table 3) and from words used in published vocabulary tests and lexical decision experiments (Studies 7 and 8). The remaining stimuli were randomly selected from the list of 920,000 words introduced in Study 5.

**Table 3:** Correlations between the newly collected human ratings and existing variables. Between brackets: the 95% confidence interval.


STUDY	N_stim_ IN COMMON	SPEARMAN CORRELATION

Schröder_12	331	.67 [.60–.72]

Schröter_17	622	.33 [.26–.40]

LinguaPix	1,489	.29 [.25–.34]

Xu_25	355	.26 [.16–.36]

Multilex word frequency	7,578	.68 [.67–.69]

Untuned GPT-FAM estimates	10,540	.85 [.85–.86]


The stimulus presentation and response collection procedures were the same as in Study 5. The only difference was that the stimulus lists consisted of nine lists of 1,075 words (1,000 new words and the 75 control words from Study 5). They were presented in 11 blocks to give participants information about their progress. Participants took between 40 and 80 minutes to complete this task, for which they received £11. In total, 115 people took part, of whom one had to be discarded because of a low correlation with the rest of the participants in that list.

A minimum of ten people saw each of the nine lists. The reliability of the lists was omega >.93. The ratings were clearly unifactorial, as shown by scree plots. BLUPS were calculated in an analysis together with the data from Study 5, to equate the lists over all 11,000 ratings. Table 3 shows the correlations with the existing human ratings.

Table 3 confirms the poor quality of the existing familiarity ratings. Only the ratings by Schröder et al. (2012) show a useful correlation of .67 with the new ratings. Of further interest is the correlation of 0.85 with the untuned GPT-FAM estimates from Study 5, which suggests that the benefit of fine-tuning is likely to be more modest than initially expected.

## Study 7: Fine-tuned GPT-FAM estimates and their validity

In Study 5 and 6 we collected human familiarity ratings for 11,000 German words. These can be used to fine-tune the GPT estimates in the same way as we did in Study 3 for AoA estimates. We used 6000 words to train the model and kept the remaining 5000 for cross-over validation.

The human ratings were multiplied by 10 and rounded off to get integer values between 10 and 70 that could be fed to GPT as feedback. We asked GPT to give us the probabilities of the 20 most frequent ratings between 10 and 70, from which a precise estimate could be calculated via the logprobs procedure (Conde et al., 2025b). The default values (auto-adjusted depending on the training dataset) were used for fine-tuning (see Conde et al., 2025b, for technical details on how to do this). The result was a fine-tuned model of ft:gpt-4o-mini-2024-07-18 using the 6000 training words within three epochs, a batch size of 11 and a learning rate multiplier of 1.8. The training process yielded a final training loss of 0.68, indicating that the new model adapted to the provided dataset while maintaining convergence.

To evaluate the improvement as a result of fine-tuning, we compared the correlations between the untuned and the fine-tuned estimates with the human ratings for the 5000 cross-validation test stimuli. The Spearman correlation increased from .85 to .91 (for the training stimuli, it increased from .86 to .98, but this is to be expected given that the stimuli were used for the fine-tuning).

A second way to check the validity of GPT-FAM estimates is to see how well they predict word knowledge in vocabulary tests. We have data for three such tests aimed at adult German-speaking participants.

The first one is the GAudI test, recently published by Bethke et al. (2025). On each trial, a word is spoken, and participants must choose among four pictures which alternative best corresponds to the spoken word. The test was tried out with university students from Marburg and the Max Planck Institute in Nijmegen, who provided useful data for 85 words. Item recognition rate varied from 30% for Ametropie [ametropia] to 100% for Spachtel [spatula].

The second vocabulary dataset we had, was for the German Peabody Vocabulary Test (Lenhard & Lenhard, 2021). This is a test with the same format as GAudI. It was presented to the same participants of dataset 1 as part of the validation of the GAudI test. Data was collected for the 72 most difficult items. Accuracy ranged from 15% for Kenotaph [cenotaph] to 100% for verzehren [consume].

The third dataset came from the NOVA test developed by Schroeders and Achaa-Amankwaa (2025). In this test, a word was presented between four non-words and participants had to select the correct alternative. For instance, Reduktion [reduction] is the correct answer in the following item: *Vintor – Dramion – Retion – Deklaven – Reduktion*. There are 110 items.

Table 4 shows the results. For each test, the correlations between accuracy and the familiarity measures were higher than the correlation between accuracy and Multilex word frequency. It should be noted that the GPT-FAM-ft estimates were overestimated because most of the stimuli were part of the training set.

**Table 4:** Spearman correlations between accuracy, Multilex, GPT-FAM, GPT-FAM-ft and human ratings for three vocabulary tests.


TEST	N_stim_	MULTILEX	GPT-FAM	GPT-FAM-ft	RATING

GAudI	85	.375	.555	.634	.669

PPVT	72	.527	.556	.622	.663

NOVA	110	.763	.760	.845	.866


## Study 8: Utility of the familiarity estimates for lexical decision performance

The quality of AI-generated familiarity estimates can also be tested by looking at how well they predict performance in lexical decisions. There were five sets of data we could use.

Brysbaert et al. (2011) tested the quality of the SUBTLEX-DE word frequencies with three datasets of lexical decision performance. The first dataset (S1) contained data for 460 words. There were three separate lexical decision studies, which we averaged for the present analysis. The second dataset (S2) included 451 words and the third dataset (S3) contained 2154 words. We could reuse these datasets to see how well accuracy rates and reaction times (RTs) correlate with Multilex word frequency and GPT-FAM estimates.

Günther et al. (2020) provided lexical decision data and reading data (gaze durations) for 1810 German compound nouns. There were two different lexical decision tasks with different non-words; only reaction times were given.

Table 5 shows the results of all the studies. GPT-FAM-FT generally outperformed Multilex. The differences were small, partly because word frequency and word familiarity ratings correlated similarly with the dependent variables. However, the latter often had missing values since not all stimuli were included in the rating studies. Therefore, the correlation coefficients between the lexical decision data and the human ratings may not be fully comparable to those with the GPT estimates.

**Table 5:** Spearman correlations for the three lexical decision experiments described in Brysbaert et al. (2011) and the three studies described in Günther et al. (2020): accuracy, reaction time (RT) and gaze duration. -- means there were not enough data because the stimuli were not part of the rating studies.


STUDY	N_stim_	MULTILEX	GPT-FAM	GPT-FAM-ft	RATING

S1_acc	460	.468	.486	.499	.445

S1_RT	460	–.700	–.656	–.650	–.539

S2_acc	451	.595	.676	.740	---

S2_RT	451	–.662	–.731	–.766	---

S3_acc	2154	.527	.594	.644	.673

S3_RT	2154	–.493	–.523	–.553	–.531

G20_RT1	1810	–.376	–.397	–.422	–.415

G20_RT2	1810	–.429	–.488	–.504	–.490

G20_gaze	1810	–.338	–.336	–.335	–.317


**Figure 12:**
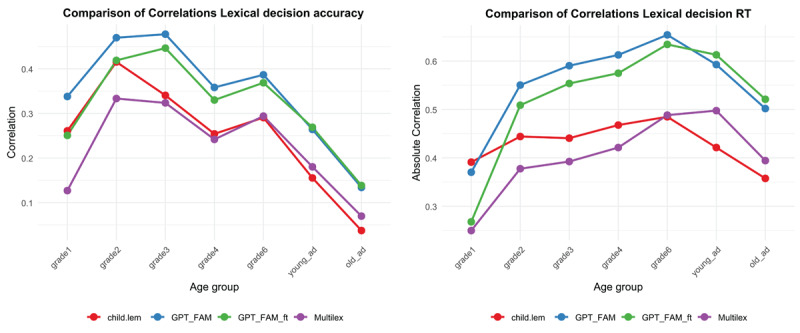
Spearman correlations with Childlex lemma frequency (red), Multilex frequency (purple), GPT-FAM (blue) and GPT-FAM-ft (green) for accuracy (left panel) and RT (right panel) in the lexical decision task of Schröter & Schroeder (2017).

A fifth set of lexical decision data available in German was published by Schröter & Schroeder (2017). They presented 1152 words to primary school children from grade 2 to grade 6, to young adults, and to old adults. Figure 12 shows the Spearman correlations of accuracy and RT with Childlex lemma frequency, Multilex frequency, GPT-FAM and GPT-FAM-ft. Surprisingly, GPT-FAM had the highest (absolute) correlation for children, whereas GPT-FAM-ft became dominant for adults. This reminds us of the fact that GPT-FAM-ft was finetuned to familiarity ratings provided by adults between 20 and 60 years old. For young children, Childlex was more informative than Multilex; for adults the reverse was true. Further good to keep in mind is that the range of frequency/familiarity was quite restricted, given that the words had to be known to primary school children. For adults nearly all words were highly familiar (see Keuleers et al., 2015, for evidence that word frequency is more informative at the high end of the scale, whereas variables like word prevalence and word familiarity are more informative at the low end of the scale).

All in all, the data from Study 8 replicated previous findings with English and Spanish stimuli (Brysbaert et al., 2025; Martínez et al., 2025a): GPT-FAM and GPT-FAM-ft most of the time outperform word frequency norms to predict lexical decision performance. The greatest gain is in the low range, since above the Zipf frequency = 4, all words are well known and familiarity no longer makes much difference (Figure 11). In this respect, familiarity resembles word prevalence, which also provides more information for low-frequency words than for high-frequency words (Keuleers et al., 2015).

## Study 9: Familiarity estimates for English cognates

In the last study we investigated to what extent the German familiarity estimates are affected by the English training of the model. GPT-4o was mainly trained with English materials, after which fine-tuning happened in German with a much smaller corpus.

Martínez et al. (2025a) proposed a way to test the extent of English-Spanish cross-language contamination. They hypothesized that if English word frequencies influenced Spanish familiarity estimates, Spanish words cognate with English words would exhibit higher familiarity ratings than control words. Their research found no such difference.

To repeat the test of Martínez et al. (2025a) for possible English-German contamination, we selected 75 word pairs, matched on Multilex frequency, word length, and part of speech. One word of each pair was an English cognate (Arm, Socke, Ozean, Habitat), the other was a control word (Zug, Säule, Münze, Ungetüm). Cognates adhered to the definition of Schepens et al. (2012) that the Levenshtein distance between the German word and its English translation must be less than half the length of the word.

**Figure 13:**
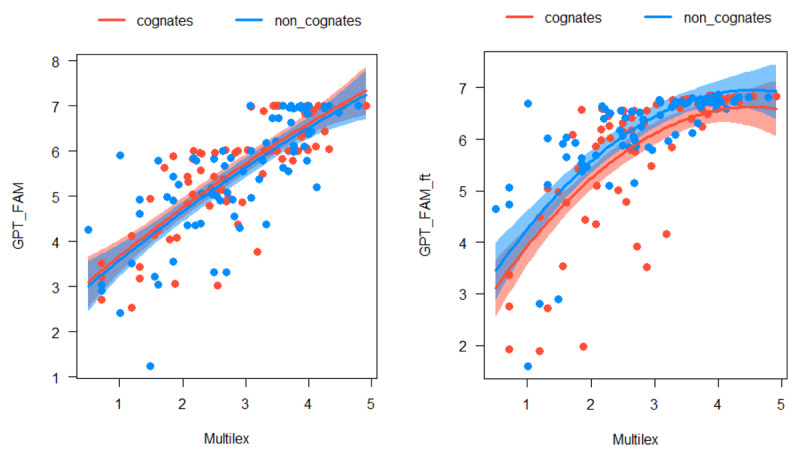
Familiarity estimates for English cognates and control words. Left panel: GPT-FAM; right panel: GPT-FAM-ft.

We collected the GPT-FAM norms for the 75 word pairs and entered them in a non-linear regression analysis with Multilex and Cognate status as predictors. Figure 13 shows the outcome. For the untuned GPT-FAM estimates, Multilex had a significant, almost linear effect (R² = .61) and Cognate status had no effect at all (t = .702, p = .484). For the fine-tuned GPT-FAM-ft estimates, Multilex had a curvilinear effect (R² = .55) and Cognate status had a slight effect in the reverse direction: Cognates were judged as slightly less familiar than their controls (t = 2.584, p = .011). Apparently, fine-tuning with German words seems to have deprecated English-looking words a bit.

## General discussion

The current article presented AI-generated estimates of five characteristics of German words that are frequently used as selection criteria in studies: word concreteness, valence, arousal, age of acquisition (AoA), and familiarity. The available data was particularly limited and fragmented for AoA and word familiarity. More extensive information was available for word concreteness, valence, and arousal, partly thanks to previous initiatives that used semantic vectors to collect AI-generated estimates for these characteristics.

The AI estimates were generated with GPT-4o-mini because, at present, this model provides better estimates than open-weight models (Conde et al., 2025a) and more recent GPT models do not appear to offer superior word features. Extensive studies have been conducted to obtain and test the estimates against existing human and AI data, so researchers can use them with confidence.

Many analyses were reported in the article, but the main conclusions are as follows:

1. GPT estimates of word concreteness correlated strongly with human ratings (Figures 1 and 2). Probably it is no longer necessary to collect ratings with human participants (except perhaps for specific words that, in the researcher’s opinion, have major theoretical implications). The LLM-based estimates are better than most other available estimates based on translation or semantic vectors, with the exception of Köper & Schulte im Walde (2016). The latter also correlated strongly with human ratings. A good strategy may be to consider both AI-generated estimates and select words for which the predictors agree, or use the difference to understand what causes the divergence.
2. GPT estimates of valence and arousal also correlated well with human ratings and with previously calculated proxies based on semantic vectors (Figures 3, 4, 5, 6). There are indications that the estimates of Köper & Schulte im Walde (2016) are better than the GPT estimates we obtained, especially for arousal. Here too, a good strategy may be to use both predictors as selection criterion or as a starting point for targeted research. We could probably improve the GPT estimates with fine-tuning, but we do not think that such fine-tuned estimates are necessary, given the existence of good alternatives.
3. GPT estimates of AoA are a good substitute for human ratings and better than the other available alternatives (Figure 7). We see a clear advantage in the finetuned estimates and recommend their use (Figures 8 and 9).
4. AI-generated estimates of word familiarity are a good alternative for word frequency norms, in particular to predict which words will be known in lexical decision tasks and vocabulary tests. Fine-tuning to adult human rating data improved the fit for adults (Figure 11 and Tables 3 and 4) but not for children (Figure 12). Fine-tuning also seemed to have a slightly negative effect on English-German cognates (Figure 13).
5. The Multilex word frequencies are better than the SUBTLEX frequencies, likely because they are based on a larger and more diverse corpus.

In general, fine-tuning improves the fit with human data (Sendín et al., 2025). At the same time, it biases the AI-generated estimates toward the people, procedure, and stimuli used for the human ratings. This was most evident with the fine-tuned familiarity estimates. As shown in Figure 12, we found that the fine-tuned estimates were more accurate for data obtained from adult participants than for data obtained from children. Fine-tuning also seemed to lower the familiarity ratings of German-English cognates (Figure 13).

Fine-tuning did not have an overall positive effect on familiarity because form-related decisions are more ambiguous than meaning-related decisions. This is evident in the entries where fine-tuning significantly lowered the familiarity ratings. Some of these entries were orthographically incorrect, such as the stimuli “Vater28,” “m-mein,” “immmer,” “Gemüse,” and “Buero.” Before fine-tuning, GPT gave these stimuli high familiarity estimates (in line with a pragmatic interpretation of the stimulus). After fine-tuning, however, these stimuli received low estimates because the system had been trained with correct German words only. The same was true for words that did not follow German capitalization rules (Warf, NICHT, Frolich, detektiv, schaze, etc.). English entries in the word list were mostly deprecated (e.g., Earth, tomato, bread, perfume, etc.), but not all (e.g., the words Goodbye, ready, forever, everybody got higher finetuned estimates).

Some other ratings that were low without fine-tuning suddenly received high estimates after fine-tuning, such as “reund”, “pielen” and “Ooooooh”. These were mostly fringe entries outside the words typically used in language studies, but the fact that they exist should be a reminder that finetuning sometimes has unintended consequences and, therefore, that it is good practice always to double-check the stimuli you work with.^4^ Differences between the estimates with and without fine-tuning are a good criterion for that. You don’t want big differences between these that cannot be explained. In case of doubt, it is always good to collect extra human ratings, which if needed can be used as (additional) fine-tuning information.

Ungrammatical entries are a problem in corpus analysis, which forms the basis for normed word lists (i.e. our list of 928,000 “words”). Although it is tempting to remove these poor entries, there are no fail-safe procedures for doing so, not even manual checking. Moreover, what may seem uninteresting at first glance may become relevant later. We have therefore decided to include all entries and let users decide which ones they want to remove.

It should be noted that ambiguous entries may further affect the outcome of search and matching algorithms because different matching procedures use different criteria. This is likely to be a more urgent problem in German than in English. For example, some procedures match words with capital letters (e.g., “Essen” and “essen” are considered the same word), while others do not. This causes a problem in German because capital letters are used for nouns but not for other parts of speech. Another German feature that is likely to cause problems is the presence of characters outside the English ASCII system. Different programs handle these characters differently. Such differences can be identified by comparing estimates collected using different procedures.

Fine-tuning generally improved the predictive validity of word familiarity estimates for words typically used in German studies (Studies 7–8). Therefore, we recommend using this variable for research involving adult participants. However, we also provide the untuned GPT-FAM estimates so that users can evaluate the impact of fine-tuning and determine its usefulness for the stimuli they are interested in.

## Data Accessibility Statement

We provide a list of GPT-FAM estimates for 928,000 word forms. These stimuli came from various sources (see the introduction to Study 5 for more details). While this is one of the most comprehensive German word lists, it is by no means complete because compound words in German are written as single words. Due to the productivity of word formation, the number of possible German compound words is virtually endless and likely exceeds 100 million (by combining more than 10,000 words). Nevertheless, the list should contain most of the words German researchers are likely to be interested in. In addition, it is rather easy to obtain LLM-based estimates for extra words using the procedures described in Conde et al. (2025b). In that case, we recommend trying a few words from our list first to ensure the model works as expected before collecting values for new words.

The familiarity list includes entries with spelling errors and symbols other than letters. Therefore, it has a column indicating whether the entry passed the German spelling check in Microsoft Office. This makes it easier for users to discard entries that are not relevant. However, this method is not foolproof either. Some irrelevant entries pass the check, such as Monopoly-Spiel, warum?, gut?, Was?, haben’, der-, and ein-. We corrected some end punctuation errors manually, but this was based on crude procedures (i.e., deciding whether the end letter was a punctuation mark or not). The MS spelling check may also exclude rare compound words that a researcher is interested in. Because the orthographic overlap between German and English words may be important to researchers, the database has a column that indicates which entries pass the MS English spelling checker.

Finally, the familiarity list includes a column with the Multilex frequencies. They are Zipf-values, meaning that values lower than 3 represent low-frequency words (with frequencies less than 1 per million words) and values higher than 4 represent high-frequency words (with frequencies higher than 10 per million words). When an entry was not present in the Multilex list, it was given a value of 0.3 (2 occurrences per billion words) if it passed the MS spelling check. This is sufficiently lower than the Zipf-frequency of 0.7 observed for words occurring once in the Multilex corpus.

Because estimates of concreteness, valence, arousal, and AoA are only useful for words that are known to participants, we provide them in a separate list with 167,000 entries. Only words with GPT-FAM estimates above 3.5 were retained, as the other words are unlikely to be known by participants typically included in psychological studies. We did some further, automatic cleaning to increase the chances that entries selected will be of interest, which is the reason why the final list is shorter than the lists discussed in Studies 1–3.^5^ At the same time, we want to warn users that we did not check individual entries. So, whenever automatic selection criteria are used, it is good to check the outcome for entries that would never be included in a dictionary. To help with this, for concreteness, valence and arousal, the estimates of Köper & Schulte im Walde (2016) are include as well.

The lists are available at https://osf.io/ghjd2/. There the reader can also find the data and the code used in the various validation studies, including the new human familiarity ratings we collected for 11,010 words.

## Footnotes

The studies are not presented in the order in which they were conducted. In chronological order, we started with the untuned word familiarity estimates (Studies 4 and 5, and parts of Studies 7–9), followed by the concreteness estimates (Study 1), the valence and arousal estimates (Study 2), and the AoA estimates (Study 3). The fine-tuned familiarity estimates were collected and analyzed last (Studies 6–9). To make the text easier to follow, the word familiarity studies have been regrouped into Studies 4–9.

Even with temperature 0, the correlation is not 1 because the output is not deterministic in OpenAI models (https://community.openai.com/t/why-does-openai-api-behave-randomly-with-temperature-0-and-top-p-0/934104). However, divergences between estimates are too small to have practical implications).

The authors thank Siri Kamp for sending them a copy of the data.

Further investigation revealed that the high familiarity estimates for the non-existent words “reund” and “pielen” were caused by the model confusing these items with Freund (friend) and spielen (play). Such confusion has been reported before (Martínez et al., 2024) and is likely related to the similarity to words that were part of the fine-tuning. The authors thank Manuel Perea for pointing out this possibility.

The full lists can be found in the data and code folders of the respective experiments, in case a researcher needs extra values for words not included in the 167K list.
